# Seroprevalence of Molluscum contagiosum Virus in German and UK Populations

**DOI:** 10.1371/journal.pone.0088734

**Published:** 2014-02-18

**Authors:** Subuhi Sherwani, Laura Farleigh, Nidhi Agarwal, Samantha Loveless, Neil Robertson, Eva Hadaschik, Paul Schnitzler, Joachim Jakob Bugert

**Affiliations:** 1 Cardiff University School of Medicine, Institute of Infection and Immunity/Medical Microbiology, Cardiff, United Kingdom; 2 Dr P N Behl Skin Institute and School of Dermatology, New Delhi, India; 3 Cardiff University Medical School, Institute of Psychological Medicine and Clinical Neuroscience, Cardiff, United Kingdom; 4 Universität Heidelberg, Hautklinik, Heidelberg, Germany; 5 Universität Heidelberg, Dept.of Infectious Diseases, Heidelberg, Germany; University of Liverpool, United Kingdom

## Abstract

Molluscum contagiosum virus (MCV) is a significant but underreported skin pathogen for children and adults. Seroprevalence studies can help establish burden of disease. Enzyme linked immunosorbent assay (ELISA) based studies have been published for Australian and Japanese populations and the results indicate seroprevalences between 6 and 22 percent in healthy individuals, respectively. To investigate seroprevalence in Europe, we have developed a recombinant ELISA using a truncated MCV virion surface protein MC084 (V123-R230) expressed in *E. coli.* The ELISA was found to be sensitive and specific, with low inter- and intra-assay variability. Sera from 289 German adults and children aged 0–40 years (median age 21 years) were analysed for antibodies against MC084 by direct binding ELISA. The overall seropositivity rate was found to be 14.8%. The seropositivity rate was low in children below the age of one (4.5%), peaked in children aged 2–10 years (25%), and fell again in older populations (11–40 years; 12.5%). Ten out of 33 healthy UK individuals (30.3%; median age 27 years) had detectable MC084 antibodies. MCV seroconversion was more common in dermatological and autoimmune disorders, than in immunocompromised patients or in patients with multiple sclerosis. Overall MCV seroprevalence is 2.1 fold higher in females than in males in a UK serum collection. German seroprevalences determined in the MC084 ELISA (14.8%) are at least three times higher than incidence of MC in a comparable Swiss population (4.9%). While results are not strictly comparable, this is lower than Australian seroprevalence in a virion based ELISA (n = 357; 23%; 1999), but higher than the seroprevalence reported in a Japanese study using an N-terminal truncation of MC133 (n = 108, 6%; 2000. We report the first large scale serological survey of MC in Europe (n = 393) and the first MCV ELISA based on viral antigen expressed in *E. coli.*

## Introduction

After the eradication of smallpox, MCV is the principal poxvirus causing human disease [1]–[4]. MCV is classified as a member of the family *Poxviridae,* in its own genus *Molluscipoxvirus* [5]. It has unique features that are distinct from other poxviruses pathogenic for humans, including smallpox and monkeypox [6]. MCV shares the highest level of amino acid (aa) similarity and unique proteins with parapoxviruses such as Orf viruses [7].

MCV infects the human skin and Molluscum contagiosum (MC) is a sexually transmitted disease, with infections occurring worldwide [1], [8]–[10]. Clinical infection is characterized by a variable number of papules, each forming a central crater filled with a waxy plug of cell debris mixed with a large numbers of virus particles. Histopathologically, MC causes a benign epidermal hyperproliferation, known as an acanthoma [11]. MC is most common in young children and teenagers. MC in immunocompromised patients results in more numerous and extensive lesions [12]. In immune-competent patients, lesion may persist for up to 12 months [11]. Spontaneous regression of MC lesions is commonly preceded by clinical signs of inflammation [13], indicating a vigorous immune response [14].

The true prevalence of MC has probably been underestimated because of the benign clinical manifestation and rare complications. Development of assays which could assist in seroprevalence studies has been hampered by unsuccessful attempts to cultivate MCV efficiently *in vitro* [15]–[18]. The viral genome was sequenced in 1996 [2].

In the first known MCV antibody study in 1952, Mitchell found three out of 14 MC patients with complement-fixing antibody to an antigen prepared from human MC lesions [19]. Shirodaria *et al*. used MCV cryostat sections in an immunofluorescence study of MCV antibodies, reporting IgM class of antibodies only in MCV patients and IgG antibody responses in 16.7% of healthy control subjects (n = 30) [20]. Only two seroprevalence studies using ELISA, have been reported; one by Konya and Thompson [21] in 1999 and another by Watanabe *et al*. in 2000 [22].

Konya and coworkers described in 1992 a virion based enzyme linked immunosorbent assay [23]. MCV virions were isolated from human lesion material. The antigen was extracted from pooled lesions of different genotypes with epidermal protein extract used as a control. Their 1999 serological survey of a healthy Australian population (n = 357) revealed an overall seroprevalence of 23% and up to 77% in MCV infected HIV negative individuals [21].

Based on MCV sequence information then available, in 1998 Watanabe *et al*. identified two immunodominant proteins of 70 and 34 kDa and mapped them to the ORFs mc133L and mc084L, respectively [24]. The proteins are homologues of vaccinia virus proteins H3L and A27L, and major antigenic peptides of the virion particle [1], [24].

Using this information they developed an ELISA, based on an N-terminal truncation of MCV virion protein MC133 produced in a Sendai virus expression system [22]. Their survey of a Japanese population of 508 subjects found mc133 specific antibodies only in 58% of patients with MC, and in only 6% of healthy controls (n = 108).

The objective of our current study was to develop a recombinant MCV ELISA using water soluble and highly antigenic truncations of MC084L expressed in *E. coli* and to establish seroprevalence in a German and a UK serum collection.

## Materials and Methods

### Ethics Statement

The study has ethical approval for the use of German tissues and sera (Ethikvotum S-091/2011 Hautklinik Heidelberg. Ethical approval was given by the Heidelberg University board in charge of ethical approvals, the ‘Ethikkommission’. Ethical approval for UK samples was part of ‘An Epidemiological study of Multiple sclerosis and other neuroinflammatory demyelinating disorders in South Wales’, 05/WSE03/111. Ethical approval was given by the Cardiff University ‘Biobank Ethics Board’. All patients provided prospective informed consent in writing upon admission. All children’s’ parents/guardians provided informed consent in writing. Class 2 GM work was notified to HSE with the project number GM 130/10.3.

### pGEX-2TK Expression of Truncated MCV –GST Fusion Proteins

The plasmid pGEX-2TK was used for expression of truncated and epitope tagged MCV ORFs mc084 (V33-G117V5), MC084 (V123-R230 StrepII), and MC133 (M1-N370 StrepII) in *E. coli* with Glutathione S-Transferase (GST) fusion protein at the N terminus. Recombinant plasmids were constructed by PCR using specific primers tailed with restriction enzyme sites (*BamHI*-*EcoRI*) and C-terminal epitope tags.

### Expression and Purification of MC084S (V123-R230) Protein

pGEX 2TK GSTmc084S (ILR#332; MC084 specific insert 107 amino acids; 14 kD) was transformed into *E. coli* BL21 (RIL^+^).Cultures were induced with Isopropyl β-D-1-thiogalactopyranoside (IPTG) and fractions analysed for fusion protein expression by SDS-PAGE and StrepII tag expression by western blotting. Cultures were incubated at 37°C for 4 h after which the cells were harvested by centrifugation at 10,000×g for 20 min and lysed by sonification in buffer B (8 M urea, 0.1 M NaH_2_PO_4_, 0.01 M and Tris-HCl, pH 8.0). Lysate containing the protein of interest was added to glutathione sepharose beads and GST-MC084S was bound to beads using batch purification. The fusion protein was cleaved using Precision protease at RT overnight. AKTA-FPLC of the resulting 14 kD sized protein was done using size exclusion Superdex S200 column (GE Healthcare).

### SDS-PAGE, Western Transfer and Immunodetection

Protein preparations were separated using denaturing sodium dodecyl sulphate polyacrylamide electrophoresis (SDS-PAGE) in NuPAGE Novex 4–12% Bis-tris Gels (Life technologies) and MOPS SDS running buffer (Invitrogen). Protein bands were visualised by staining with 0.01% Coomassie Brilliant Blue R-250. For immunodetection proteins prepared by SDS-PAGE were electrotransferred onto nitrocellulose and probed with Strep MAB Classic HRP conjugate (IBA). Detection by chemiluminescence was performed using Super Signal West Pico Chemiluminescent Substrate (Thermo Scientific) according to the manufacturer’s recommendations.

### Human Serum/Tissue Samples

314 serum samples and lesion material from patients with molluscum contagiosum were collected at University Hospital Heidelberg, Germany, between 2007–2011. 79 UK sera samples were collected at Cardiff University. Twelve serum samples were collected from MCV patients (10 from Dr P N Behl Skin Institute and School of Dermatology, New Delhi, India; two from UK; aged 2–62 years) as diagnostic specimens.

### MCV Direct Binding ELISA

Ninety six well Maxisorp ELISA plates (Nunc) were coated with 3 µg/ml of FPLC purified recombinant truncated MC084S (aa 123–230) protein per well in 100 µl of 0.05 M carbonate-bicarbonate buffer (pH9.6) and incubated at 37°C for 2 h and then overnight at 4°C. Plates were washed with PBS and blocked with 5% skim milk. Test sera, diluted 1∶100 in dilution buffer, and were coated across the plate (100 µl/well). The plates were incubated at 37°C for 2 h and washed ten times with PBS-T. Secondary anti-human IgG conjugated to horseradish peroxidase (GE Healthcare), diluted 1∶2000 in dilution buffer was subsequently added (100 µl/well). After incubation at 37°C for 2 h the plate was washed ten times with PBS-T and 100 µl of BD OptEIA™ substrate reagents (BD Biosciences) was added to each well. 50 µl of 1 M H_2_SO_4_ was used to stop the enzyme reaction after 20 min incubation at RT. The OD of the reaction product was read at 450 nm on an FLUROSTAR OPTIMA - ELISA plate reader (BMG Labtech).

### Plate Description

42 serum samples were tested in duplicate on each plate along with a panel of four control sera consisting of two negative and two positive as well as four blanks, all in duplicate. The results were expressed as δODU (δODU = mean of duplicate wells minus mean of the blank wells).

### ELISA Performance

Plate to plate variation was monitored by comparing the control panel results between the different wells of the same plate; same sera samples run on different plates on the same day as well as on different days.

### Immunofluorescence and Immunohistochemistry

Paraffin embedded sections were deparaffinized and rehydrated. Dako Cytomation Envision®+Dual Link System-HRP (DAB^+^) kit (Dako) was used as per manufacturer’s instructions. For staining of tissue with human sera, ECL Anti-human IgG (1∶2000) (GE Healthcare) was used. Staining was completed with Mayers haematoxylin and eosin counterstaining. All sections were analysed using an Olympus BX51 light microscope. Vaccinia virus infected HaCaT cells were grown on glass coverslips and fixed with 3% paraformaldehyde for 10 min, followed by staining with human serum antibodies (1∶100) and an anti-human AlexaFluor 488 secondary antibody (Invitrogen).

### Statistical Analysis

Serological data was stratified by age or diagnosis. Statistical significance of differences between the ELISA responses of different groups was assessed by one way ANOVA. Tukey post hoc anova was used to identify and compare statistically significant means and differences of different groups.

Additional information on material and methods is shown in supporting information.

## Results

### Selection of MC084 Antigen, Cloning and Purification

**Figure 1:**
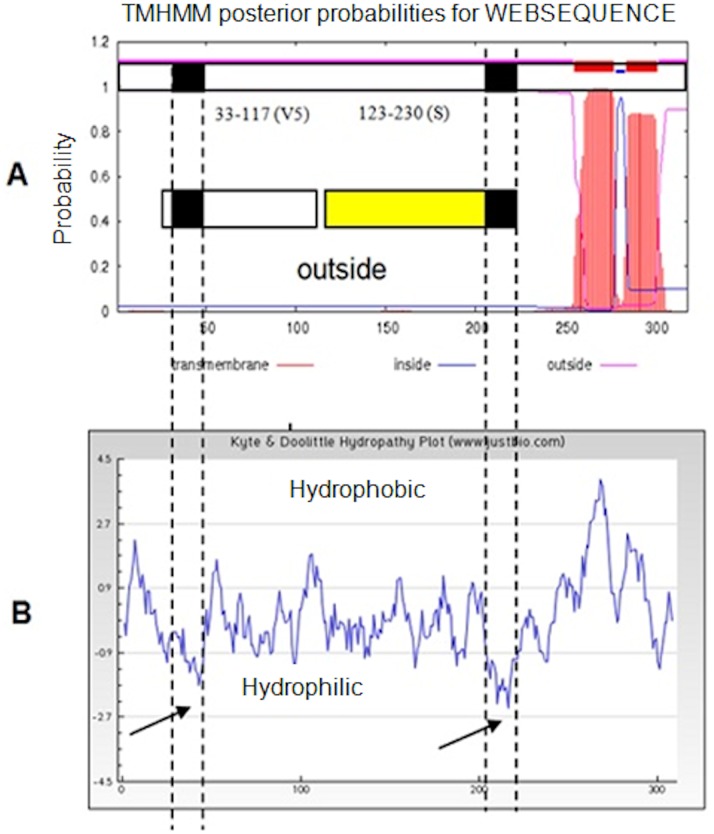
Bioinformatics. (A) Transmembrane plot (TMHMM Server v.2.0) [25] of mc084 amino acids 1–318; (B) hydropathy plot of MC084 protein with predicted high hydrophilic/antigenic regions indicated by black boxes. The full length ORF (MC084 1–318; predicted molecular weight 34.2 kD; shown on top) was cloned into vRB12 using specific primers tailed with restriction enzyme sites *BamHI*-*HindIII*) and C-terminal StrepII epitope tag. The resulting plasmid p319 was sequenced and the recombinant vaccinia virus v319 isolated on BSC-1 cells using the plaqueless mutant system [26]. N- and C-terminal (in yellow) truncations were subcloned from the original full length MCV gene into pGEX-2TK for overexpression in *E. coli* BL21 (RIL^+^). TMHMM was used to determine transmembrane regions [26] whereas the Kyte-Doolittle plot was used to identify hydrophilic regions with predicted high antigenicity [27].

Amino acid sequences of MC084 (298 aa, 34 kD) were analysed to determine overall homology with related proteins in the GenBank and identify transmembrane regions and region of high hydrophilicity/high antigenicity. Two transmembrane regions predicted in the C-terminal end of the protein [26], were excluded to avoid solubility issues in the *E. coli* expression system (Figure 1A). Of the remaining amino acids, a N-terminal region (V33-G117) and a C-terminal region (aa V123-R230), both containing one region of high hydrophilicity in the Kyte–Doolittle plot (Figure 1B) [28] were further analysed for subcloning.

**Figure 2:**
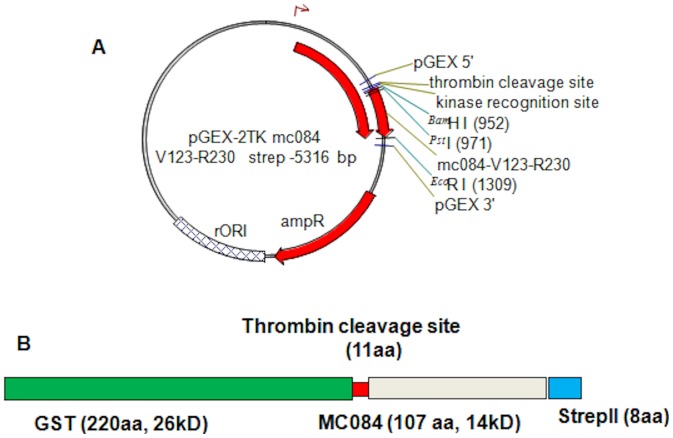
pGEX 2TK construct. (A) Schematic of recombinant plasmid p332 with a MC084 specific insert of 107 amino acids (V123-R230); predicted molecular weight 14 kD (B) Schematic of fusion protein of GST (green), followed by Thrombin kinase site (red), MC084 V123-R230 (grey), and strep II tag (blue); predicted antigenic site (black) (C) Western blot giving 40 kD GST fusion protein GST-MC084S (V123-R230) detected using Strep MAB-Classic HRP conjugate (IBA-lifesciences). Vector NTI (vNTI) was used to produce virtual molecules and schematic diagrams of constructs prior to molecular cloning (InforMax, Inc).

The C-terminal truncation of mc084 (V123-R230, predicted MW 14 kD), comprising 107 aa, has the lowest homology to orthopoxvirus proteins and contains a region of high antigenicity (218-NELRGREYGASLR-230) with no significant homology to vaccinia/cowpox virus. The C-terminal truncation MC084 (V123-R230) was then subcloned into the pGEX-2TK vector (Figure 2A) with a Strep II tag and in frame with glutathione S-transferase separated by a thrombin kinase site (Figure 2B) and overexpressed as a GST fusion protein in codon optimized *E. coli* (BL21 RIL^+^).

**Figure 3:**
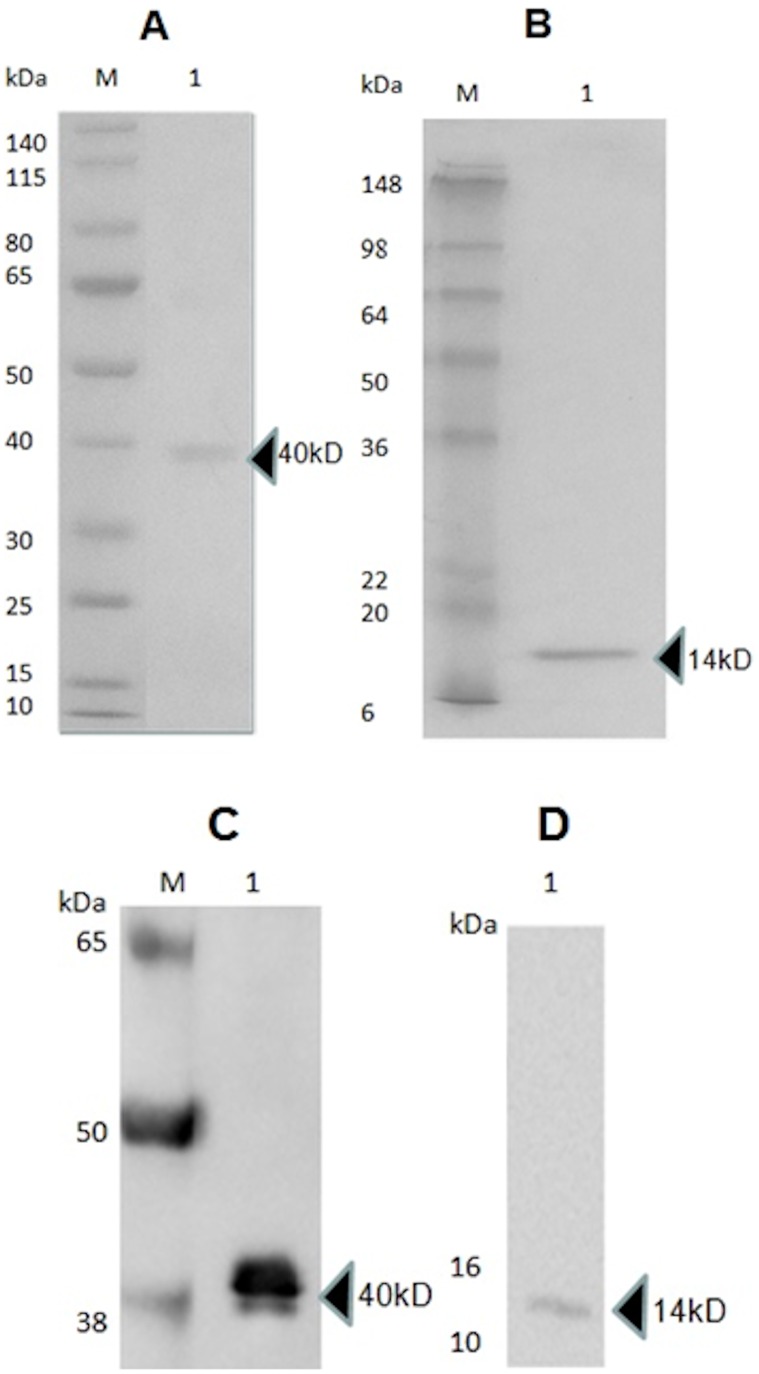
Protein purification. Characterisation of over expressed recombinant fusion protein GST-MC084S and FPLC purified recombinant MC084S protein by SDS-PAGE and Western Blot. M: Molecular weight markers expressed in kDa. (A) Over-expressed 40 kDa Recombinant GST-MC084S fusion protein separated in a 4–12% Bis-Tris gel. (B) FPLC purified 14 kDa protein separated in a 15% Bis-Arylamide gel. Both gels were stained with Coomassie Brilliant Blue R-250. (C) GST-MC084S fusion protein after transfer to nitrocellulose (D) FPLC purified MC084S. The membranes were probed with Strep MAB-Classic HRP conjugate (IBA-lifesciences). Arrow heads indicate the locations of proteins.

The GST fusion protein was identified with an apparent molecular weight (MW) similar to the predicted MW of 40 kD in IPTG induced cultures (Figure 3A) and was absent in the uninduced cultures. The protein was protease cleaved and the C-terminal truncation of MC084 with an apparent MW of 14 kD was further purified via FPLC using a size exclusion Superdex S200 column (Figure 3B). The Strep II tag was identified in Western Blot in both the fusion protein and the cleaved MC084 (V123-R230)-Strep II truncation (Figures 3 C and D). Additional data on antigen selection and optimization can be found in Figures S1 and S2.

### ELISA Sensitivity, Cut-off, and Specificity

MC084S (V123-R230) antigen coated ELISA plates were produced as described in material and methods. Serum samples were diluted 1∶100.

**Figure 4:**
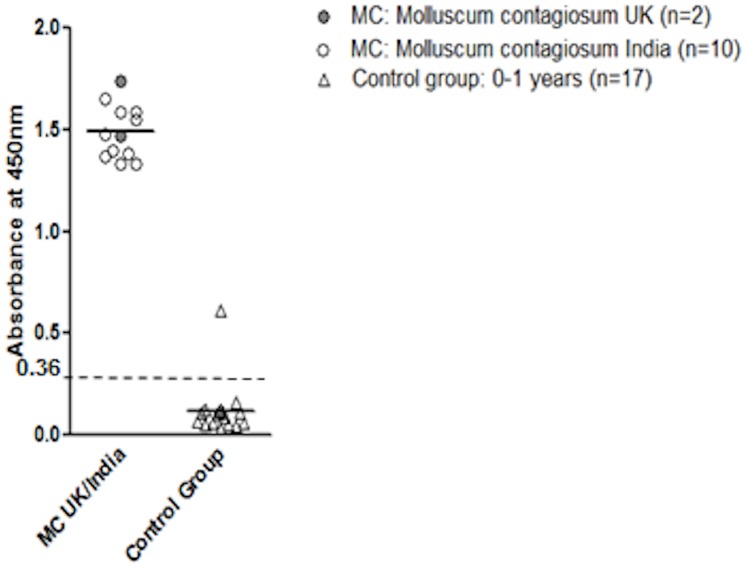
Sensitivity. Absorbance plot of twelve sera from patients clinically diagnosed with MCV (India n = 10; UK n = 2; control group of 0–1 year old individuals n = 17).

To establish sensitivity a panel of 12 sera from patients with known and clinically active MCV was first screened in comparison to sera from 0–1 year old individuals from the neonatal screening program of the Heidelberg University Clinics. In the group of sera from patients with diagnosed Molluscum contagiosum (n = 12) the ELISA gave high readings for all (median δODU 1.5), with the most recent sample from Cardiff (CF2012-1) giving the highest (Figure 4). The control group of seventeen neonates from the Heidelberg University Clinics showed low readings with a mean of 0.1 δODU as shown in Figure 4, with one outlier (0.61 δODU). The confidence interval for the difference between positive and negative control groups was highly significant (Figure 4). Sensitivity was 100% for MC patients.

The cut-off for ELISA was calculated based on 66 sera from infants seen in the neonatal unit aged 0–1 years. The mean of δODU readings was 0.12043 and the SD was 0.08300. In comparison the mean δODU for 12 MCV infected patients was 0.833 and the S.D. 0.571. The infant group was used to define negativity with the upper limit being the mean δODU plus 3 SD (i.e. 0.36). Assuming that these values are indicative of a negative response to the recombinant protein, we defined a positive antibody response as being a value greater than mean plus 3 SDs i.e. δODU 0.36. Two more outliers were identified in this group (δODU 0.36 and 0.35). The MCV status of these subjects (aged 2 months, 9 months and 11 months) could not be determined. Inter-well, intra-assay and inter-assay variability was found to be 3%, 5.2% and 6.7%, respectively.

In order to establish ELISA specificity, human MCV infected tissue section obtained from the Heidelberg University Dermatology Unit were tested with high and low titre sera form our serum collections (Figure S3). Reactivity of high titre sera are shown on the left (Figure S3 1A, B/transverse section-perpendicular to level skin and 2A, B/plane section-parallel to level skin). High titre sera show strong MC084 specific staining of cellular debris and MC bodies extruded from and in the centre of lesions, as well as infected cells in lobules extruding infectious virus into the centre of the lesion and upwards. Molluscum rich lipid debris areas are well preserved in these lesions. There is much weaker staining with low titre sera (same lesions in transverse and plane sections; Figure S3 1C, D and 2C, D).

**Figure 5:**
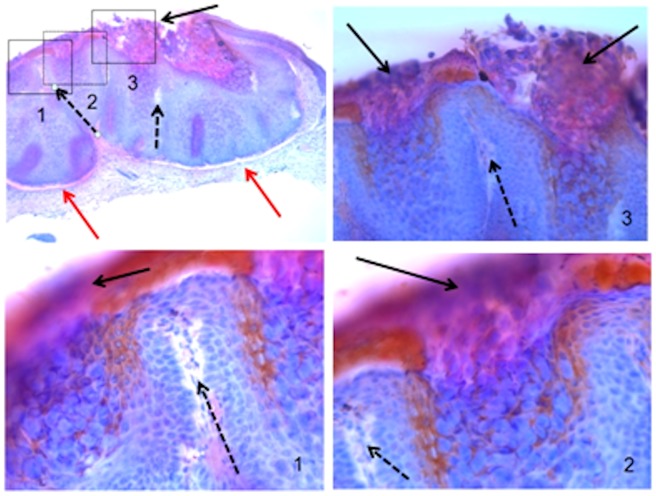
Tissue stain details. Microscopy (4×) of a Molluscum contagiosum lesion section (17315/11) stained with MC patient positive serum (CF2012-1) and haematoxylin-eosin counterstain (upper left hand corner). Three insets showing details at various magnifications [inset 1-(10×), inset 2-(20×) and inset 3-(20×)].

Specific reactivity (MCV positive UK patient CF2012-1) is demonstrated in more detail in Figure 5. The section shows a dome-shaped contour with cup shaped lesions with central invagination, representing a typical MCV lesion consisting of two inverted lobules of hyperplastic squamous epithelium (red arrows) with several sub-lobules. The MC lesion shows acanthosis with the appearance of intraepidermal lobules with enlarged basophilic nuclei filled with cellular debris and molluscum bodies (black arrows). Intraepidermal lobules are separated by septa consisting of compressed dermis (dotted arrow). MCV inclusion bodies stain strongly golden-brown with the human polyclonal serum CF2012-1 taken from a patient with clinical MCV infection. The stain is confined to areas where MCV cores, mature and released virus particles would be expected. In a number of tissue sections stained, the pattern was repeatable and sensitive to tissue preparation. Interestingly, the debris areas filled with mature MCV particles and lipid debris are also sensitive to removal by xylene/ethanol treatment of paraffin sections. The area’s most consistently stained are the suprabasal and spinous layers.

**Figure 6:**
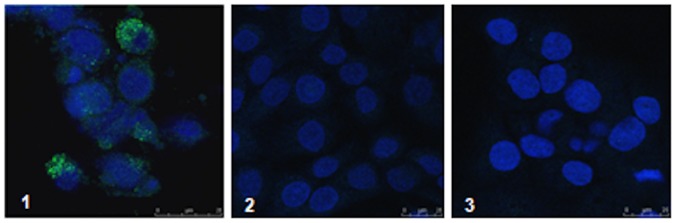
HaCaT Immunofluorescence. (A) HaCaT cell culture infected with recombinant vaccinia virus expressing MC084S (v319). Reactivity of high titre human serum HDV0901071 and secondary antibody AlexaFluor 488 (Green) goat anti-human IgG (H+L). (B) HaCaT cell culture infected with recombinant vaccinia virus expressing MC084S (v319). Reactivity of low titre human serum HDV0900040 and secondary antibody AlexaFluor 488 (Green) goat anti-human IgG (H+L). (C) Mock infected cells. Reactivity of high titre human serum HDV0901071 and secondary antibody AlexaFluor 488 (Green) goat anti-human IgG (H+L). Nuclei are stained with DAPI (Hoechst) and shown in blue. Samples were analysed for fluorescence emission properties by using confocal scanning laser microscopy Leica TCS SP2 AOBS.

To further establish antigen specificity we also infected human HaCaT keratinocytes with a vaccinia virus expressing full length mc084 (v319; aa 1 to 318) as shown in Figure 6. Infected keratinocytes were tested with the high titre serum HD V0901071. Virus infected cells show a vesicular stain similar to an endosomal/lysosomal pattern. Uninfected cells show no background signal, indicating the human polyclonal does not recognize keratinocyte antigens in cultured HaCaT cells.

Antigen optimization and comparisons are described in supporting information (Figures S1 and S2).

### ELISA Population Studies

**Figure 7:**
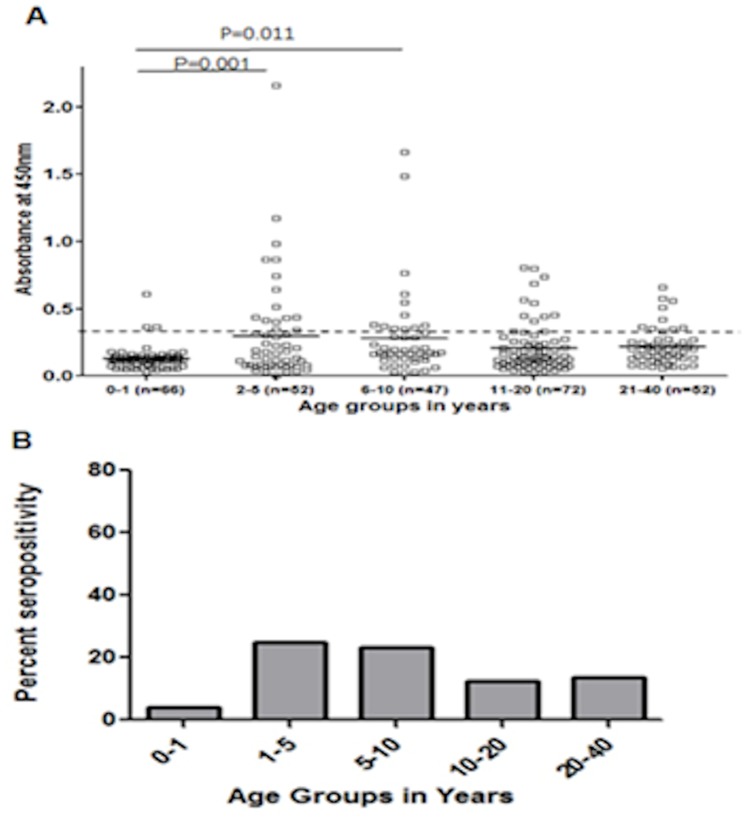
German seroprevalence. Distribution of anti-MC084S antibodies in a German population tested by direct binding ELISA (A) Serological responses to MCV antigen MC084 in a German population (n = 289; ages 0–40 years) expressed as the δODU value of an individual serum sample. The horizontal bar within each group represents the median absorbance measurement. (B) Percent seropositivities in different age groups after cut–off of 0.36 (i) 0–1 years (4.5%), (ii) 2–5 years (25%), (iii) 6–10 years (23.4%), 11–20 years (12.5%), and 21–40 years (13.5%).

Sera from 289 individuals aged 2 months to 40 years (median age 21 years) were randomly selected from frozen ‘normal control sera’ collected at the University of Heidelberg, Germany, and tested for the presence of anti-MC084S (aa 123–230) antibodies (Figure 7 A). Healthy subjects are divided into groups on the basis of age: 0–1 years (n = 66), 2–5 years (n = 52), 6–10 years (n = 47), 11–20 years (n = 72) and 21–40 years (n = 52). The reactivity in infants was significantly lower than in other groups. Based on the minimum cut-off value of δODU 0.36, 43 (14.8%) sera of the 289 sera from a representative healthy German population tested positive in the MC084S (123–230) ELISA. Positive antibody responses in the age groups were as follows: 4.5% (n = 3) 0–1 year olds, 25% (n = 13) in 2–5 year olds, 23.4% (n = 11) in 5–10 year olds, 12.5% (n = 9) in 10–20 year olds and 13.5% (n = 7) in 20–40 year olds (Figure 7 B). A one way anova was used for preference differences between the different age groups. The test statistic (F value) is 4.587 and the p value is 0.001. Further post hoc analysis was done using Tukey test to identify and measure statistically significant difference between groups of data as pairs. From the multiple comparisons it can be concluded that there is a sharp increase in positive sera responses between 0–1 year olds and 2–5 year olds which are statistically significant at p value = 0.001. The differences in the sera responses between 0–1 year olds and 6–10 year olds are also statistically significant (p = 0.011). Differences in sera responses between all other groups are not statistically significant. The results of the serological survey in members of the German populations are shown in Table 1.

**Table 1:** Summary of seroprevalences in German and UK populations.

Groups	Total sera	Positive sera ≥ cut-off = Mean+3*SD (0–1 yr+outlier) 0·36
**German sera**		
0–1 years	66	3 (4·5%)
2–5 years	52	13 (25%)
6–10 years	47	11 (23·4%)
11–20 years	72	9 (12·5%)
21–40 years	52	7 (13·5%)
	289	**Seropositivity in healthy subjects**14·87%
Psoriasis	10	2 (20%)
SLE*	3	1 (33·3%)
Autoimm†	12	2 (16·6%)
**UK sera**		
Healthy Humans	33	10 (30.3%)
PPMS#	9	1 (11·1%)
RRMS<$>\scale 80%\raster="rg1"\<$>	37	6 (16·2%)
Total	393	65 (16.5%)

*SLE – Systemic Lupus Erythematosus.

† Autoimm. – General autoimmune conditions.

# PPMS – Primary progressive multiple sclerosis.

^<$>\scale 80%\raster="rg1"\<$>^RRMS – Relapsing remitting multiple sclerosis.

We analysed 25 patients (8–40 years of age) with dermatological conditions such as Systemic lupus erythematosus (n = 10), Psoriasis (n = 3) and general autoimmune conditions (n = 12), including patients with Autoimmune haemolytic anaemia, Autoimmune cerebilitis and Autoimmune hepatitis diagnosed at the University of Heidelberg. The findings are summarized in Table 1. MCV seroprevalence is above the average rate in skin specific autoimmune conditions, but similar in general autoimmune conditions.

**Figure 8:**
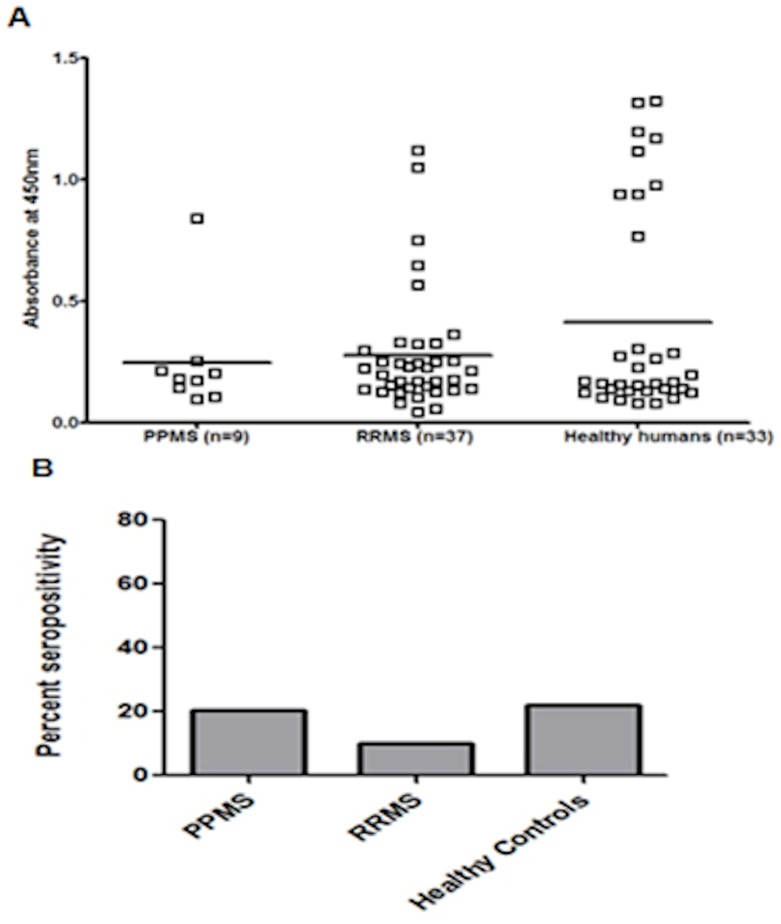
UK seroprevalence. Distribution of anti-MC084S antibodies in a UK population tested by direct binding ELISA. (A) Serological responses to MCV antigen MC084S (V123-R230) in UK population (n = 79) expressed as the δODU value of an individual serum sample in different groups (i) Primary progressive multiple sclerosis (PPMS; n = 9), (ii) Relapsing remitting multiple sclerosis (RRMS; n = 33) and (iii) Healthy humans (n = 37). The horizontal bar within each group represents the median absorbance measurement (B) Percent positivity in individual groups for MC084S after cut-off of 0.36 (i) PPMS (11.1%), (ii) RRMS (16.2%) and (iii) healthy humans (30.3%).

79 serum samples from a UK population (aged 21–40 years; median age 27 years) were analysed which had been collected as part of a study on Multiple sclerosis (MS) at Cardiff University. These subjects were grouped as Primary progressive multiple sclerosis (n = 9), relapsing remitting multiple sclerosis (n = 37) and healthy humans (n = 33) (Figure 8A).

Using the same cut-off of 0.36, MCV antibodies were detected in 10 of 33 healthy UK serum samples (30.3%). In patients with primary progressive multiple sclerosis seroprevalence was 11.1% (n = 1/9), as compared to 16.2% (n = 6/37) in patients with relapsing remitting multiple sclerosis (Figure 8B). A one way anova was used for preference differences between the different groups. The test statistic (F value) is 1.756 and the p value is 0.180. Further post hoc analysis was done using Tukey test to identify and measure statistically significant difference between groups of data as pairs. From the multiple comparisons it can be concluded that differences in sera responses between groups are not statistically significant. An overall gender ratio (M: F) of 1.4∶1 (183∶131) was found in the German serum collection, as compared to 1∶2.1 (25∶54) in the UK population. The results of the serological survey in members of all UK populations are shown in Table 1.

## Discussion

We describe here for the first time a seroepidemiological study of MCV in Europe, the largest survey reported so far (n = 393) and the first MCV ELISA based on viral antigen expressed in *E. coli*.

Previously reported MCV ELISAs used antigen from human lesion material or Sendai virus expressed N-terminal amino acid sequences of MC133, raising issues with background skin antigens and posttranslational antigen processing. To improve water solubility and provide an expression platform more suitable for commercial production of a MCV ELISA, we decided to use hydrophilic antigenic regions of MC084 expressed in *E. coli*. On the basis of previous work by Watanabe *et al.* [22] and our own homology analyses we chose a C-terminal truncation of MC084 (V123-R230), upstream of A238-Q298 previously found non-reactive in ELISA by Watanabe [22], as our candidate ELISA antigen. Our choice of antigen minimizes the possibility of cross reactivity with vaccinia virus specific antibodies, exclude the membrane spanning domains of mc084, but include a possible major antigenic site, identified by hydrophilicity plotting (MC084 N218-R230).

The ELISA is sensitive (100%) and specific, with low inter- and intra-assay variability. This is in comparison to the lower sensitivities of 71% and 58%, in the ELISAs reported by Konya *et al.* [21] and Watanabe [22], respectively. We have determined specificity in MCV tissue sections, similar to Konya *et al.* [21]. To determine specificity quantitatively, a collection of sera would be needed.

We have calculated cut-off for our ELISA to include outlier results from our neonatal control group. The MC status of the outliers could not be determined, as the data was anonymised.

Any comparisons of our findings with previous ELISA results must be fundamentally flawed, because different antigen and expression systems were used. However, no other data are available, so with the above reservations, we compared the findings of our serological survey to results reported for Northern Ireland and two previous ELISA studies in Australia and Japan [20], [21]–[22]. We find an overall seropositivity in a general German population of 14.8% and 30.3% in the UK. This correlates well with previous findings of 16.7% in Ireland (n = 30; IgG responses) [20], 23% in an Australian population [21] and less so with 6% reported in a Japanese survey [22].

The age profile determined using the MC084 ELISA correspond well with our understanding of the natural history of MCV infections, with low exposure of very young children and a high prevalence among toddlers and preschool children, where MCV smear infections is most likely to be transmitted among larger numbers of children. Our data confirm previously reported findings of stronger antibody responses in acute MC [21], mostly in the 2–10 age group [25], [28], with waning antibody levels being detectable as the population ages. This would suggest very little re-exposure in older age groups.

In contrast to Konya *et al*. [21], who report a very high seropositivity rate in their 0–6 month old population of 31%, explaining this with maternal antibodies, our data do not indicate a high seropositivity rate in very young children. Seroprevalence with the mc084 (V123-R230) ELISA is below 5% in 0–1 year olds and only increases in the age group of 2–5 year olds, not exceeding 25%. Watanabe *et al*. explained their low overall seropositivity (n = 108, 6%) [22] in healthy subjects in comparison to the prior Australian study (n = 357; 23%) [21], with their mc133 ELISA failing to pick up sera with mc084 antibodies as shown in immunoblots, indicating that mc133 may not be the best choice of antigen, underestimating seroprevalence.

The findings in immunocompromised patients and patients with skin and other inflammatory disorders indicate an increased seroprevalence in skin disorders, and a decrease in generally or therapeutically immunocompromised populations, but lack statistical power because of low sample numbers. The gender ratios calculated, indicate a higher seroprevalence (2.1 fold) in females than males of in the UK serum collection, but a lower ratio in the German collection.

In summary, we propose MC084 (V123-R230) is a suitable antigen for MCV serological surveys when expressed in *E. coli*. It includes a probable highly antigenic site at amino acid position N219-R230. Importantly, the MC seroprevalence of 14.8% in our German population is a threefold increase over the reported incidence of MC in a comparable Swiss population of 4.9% [29], supporting the notion, that MC is an underreported infection. The assay will allow further investigations into the seroprevalence of MCV in other geographical areas, including the US, China, Japan and Australia.

Ongoing work includes possible use of a subpeptide of MC084 (N218-R230) comprising only the highly antigenic site for a capture ELISA and T cell studies, and the development of an IgM MC084 (V123-R230) ELISA. We are also in the process of investigating the MC084 (V123-R230) peptide for its potential to compete with MCV/VACV entry in a MCV/VACV reporter assay [30].

## Supporting Information

Figure S1 **MC084 antigen optimization.** The figure shows the antigenicity of MC084S (aa123–230) as determined by direct binding ELISA using high titre human serum (HD V0901071). (A) Saturation was achieved at 3 µg/ml. (B) A maximum of 80% inhibition of anti-serum antibodies with MC084S as inhibitor was observed whereas negligible inhibition was observed with BSA and human IgG.
(TIF)

Figure S2 **Comparison of antigen reactivity.** The N-terminal truncation of MC084 i.e. MC084v5 (33–117), C-terminal truncation of MC084 i.e. MC084S (123–230), N-terminal truncation of MC133 i.e. MC133S (1–370) and GST tested as uncleaved fusion proteins on a GST affinity plate to compare antigen affinity and seroreactivities. The relative absorbance of individual sera was the same against all antigens tested with only minimal differences in absorbance.In direct antigen comparison there was no significant difference between truncations of mc084 and mc133, and no serum showed prevalent reactivity against one or another of the antigen used. A strep tag was used for detection of recombinant antigen in western blots. The tag did not interfere with ELISA results in a serum study of 149 serum samples.
(TIFF)

Figure S3 **Tissue staining with high and low litre sera.** Tissue sections stained with high (HD V0901071 (**1A, B**), HD V0903005 (**2A, B**) and low titre sera (HDV0900471 (**1C, D**), HDV0900040 (**2C, D**) in two magnifications (4x and 10x). High titre sera stained the spinous layers as well as cellular debris and MC bodies in and around the intraepidermal lobules golden-brown. The same section stained with low titre sera as determined in MC084S ELISA showed much reduced or no reactivity in the same tissue areas.
(TIFF)
