# Supplementary figures and images for: Seroprevalence of Molluscum contagiosum Virus in German and UK Populations
Source: PLoS One. 2014 Feb 18;9(2):e88734. doi: 10.1371/journal.pone.0088734 (PMC3928281; doi:10.1371/journal.pone.0088734)

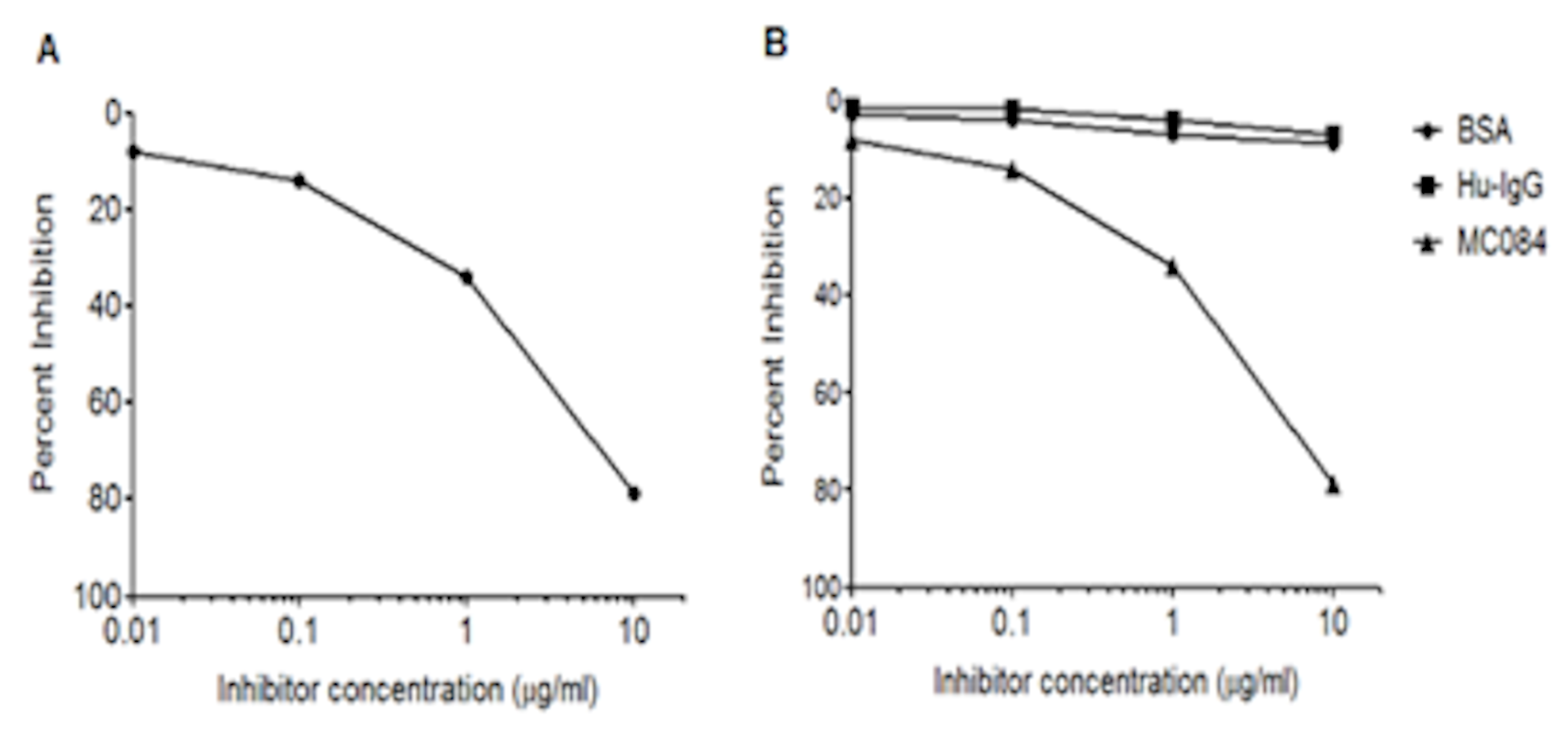

Supplement: Figure S1 — MC084 antigen optimization. The figure shows the antigenicity of MC084S (aa123–230) as determined by direct binding ELISA using high titre human serum (HD V0901071). (A) Saturation was achieved at 3 µg/ml. (B) A maximum of 80% inhibition of anti-serum antibodies with MC084S as inhibitor was observed whereas negligible inhibition was observed with BSA and human IgG. (TIF) [file pone.0088734.s001.tif]

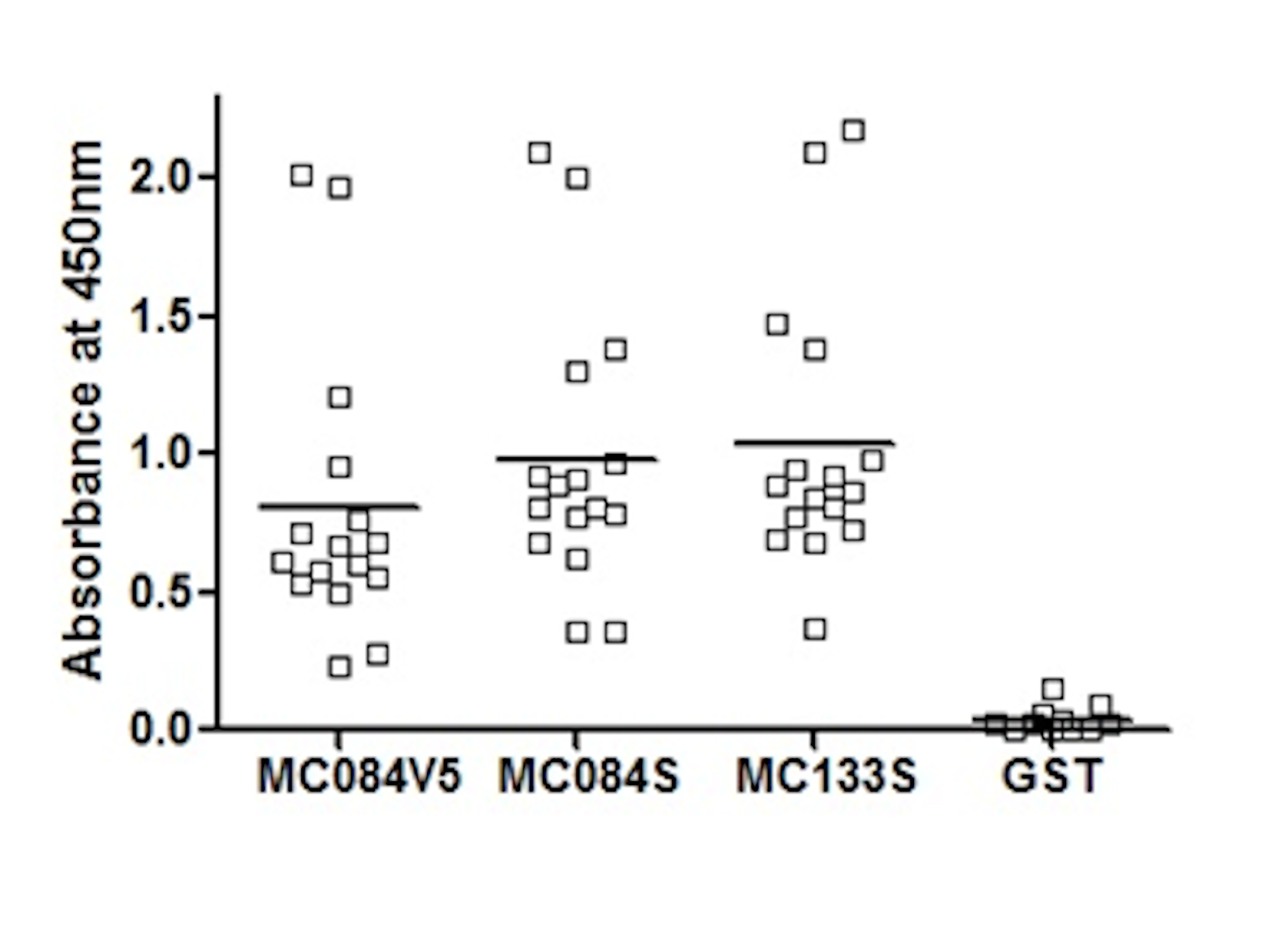

Supplement: Figure S2 — Comparison of antigen reactivity. The N-terminal truncation of MC084 i.e. MC084v5 (33–117), C-terminal truncation of MC084 i.e. MC084S (123–230), N-terminal truncation of MC133 i.e. MC133S (1–370) and GST tested as uncleaved fusion proteins on a GST affinity plate to compare antigen affinity and seroreactivities. The relative absorbance of individual sera was the same against all antigens tested with only minimal differences in absorbance.In direct antigen comparison there was no significant difference between truncations of mc084 and mc133, and no serum showed prevalent reactivity against one or another of the antigen used. A strep tag was used for detection of recombinant antigen in western blots. The tag did not interfere with ELISA results in a serum study of 149 serum samples. (TIFF) [file pone.0088734.s002.tif]

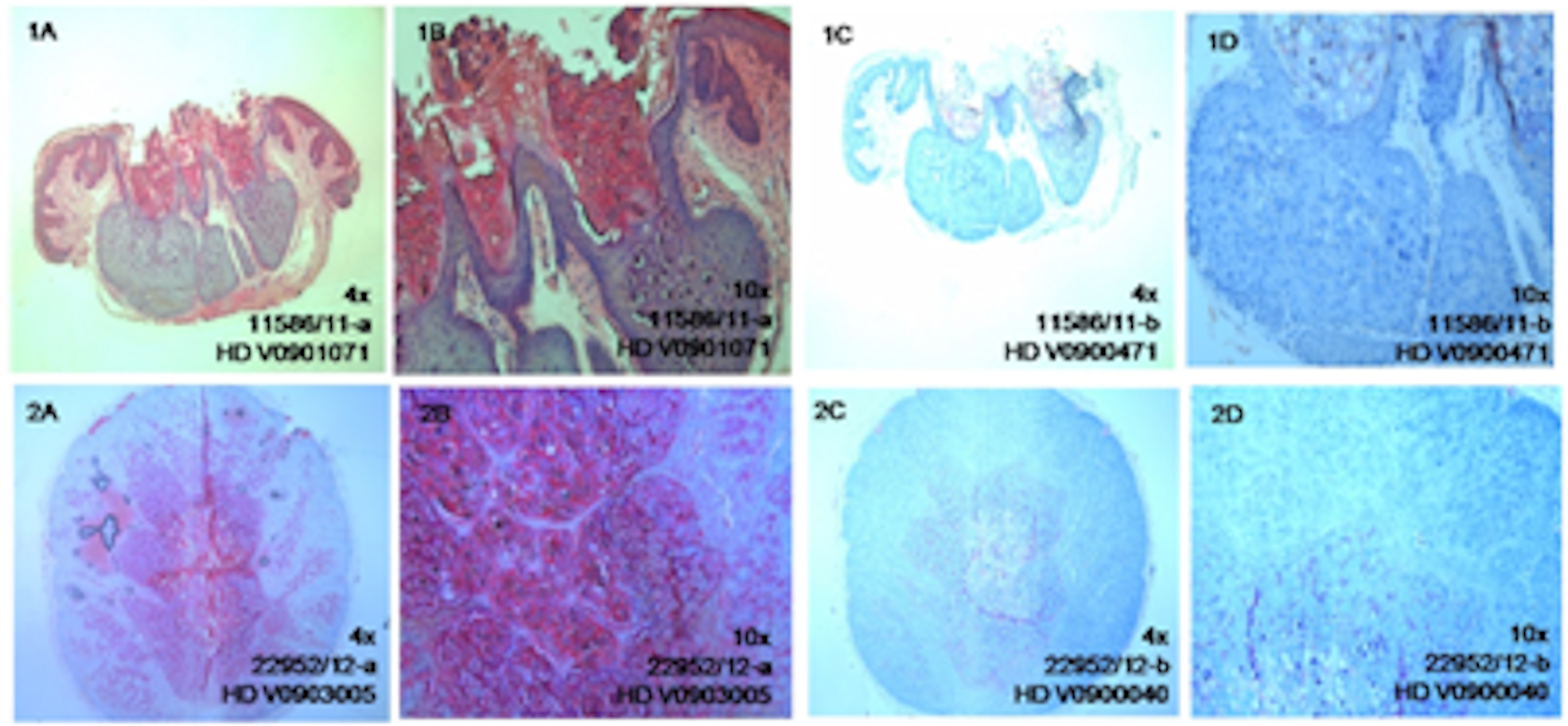

Supplement: Figure S3 — Tissue staining with high and low litre sera. Tissue sections stained with high (HD V0901071 (1A, B), HD V0903005 (2A, B) and low titre sera (HDV0900471 (1C, D), HDV0900040 (2C, D) in two magnifications (4x and 10x). High titre sera stained the spinous layers as well as cellular debris and MC bodies in and around the intraepidermal lobules golden-brown. The same section stained with low titre sera as determined in MC084S ELISA showed much reduced or no reactivity in the same tissue areas. (TIFF) [file pone.0088734.s003.tif]
